# Selective inhibition of soluble tumor necrosis factor signaling reduces abdominal aortic aneurysm progression

**DOI:** 10.3389/fcvm.2022.942342

**Published:** 2022-09-16

**Authors:** Silke Griepke, Emilie Grupe, Jes Sanddal Lindholt, Elizabeth Hvitfeldt Fuglsang, Lasse Bach Steffensen, Hans Christian Beck, Mia Dupont Larsen, Sissel Karoline Bang-Møller, Martin Overgaard, Lars Melholt Rasmussen, Kate Lykke Lambertsen, Jane Stubbe

**Affiliations:** ^1^Department of Cardiovascular and Renal Research, Institute of Molecular Medicine, University of Southern Denmark, Odense, Denmark; ^2^Elite Research Centre for Individualized Medicine in Arterial Diseases (CIMA), Odense University Hospital, Odense, Denmark; ^3^Department of Cardiothoracic and Vascular Surgery, Odense University Hospital, Odense, Denmark; ^4^Department of Clinical Biochemistry and Pharmacology, Odense University Hospital, Odense, Denmark; ^5^Department of Neurobiology, Institute of Molecular Medicine, University of Southern Denmark, Odense, Denmark; ^6^Department of Neurology, Odense University Hospital, Odense, Denmark; ^7^BRIDGE—Brain Research—Inter-Disciplinary Guided Excellence, Department of Clinical Research, University of Southern Denmark, Odense, Denmark

**Keywords:** cardiovascular disease, abdominal aortic aneurysm, tumor necrosis factor inhibitor, inflammation, translational research, vascular inflammation

## Abstract

**Background:**

Tumor necrosis factor (TNF) is pathologically elevated in human abdominal aortic aneurysms (AAA). Non-selective TNF inhibition-based therapeutics are approved for human use but have been linked to several side effects. Compounds that target the proinflammatory soluble form of TNF (solTNF) but preserve the immunomodulatory capabilities of the transmembrane form of TNF (tmTNF) may prevent these side effects. We hypothesize that inhibition of solTNF signaling prevents AAA expansion.

**Methods:**

The effect of the selective solTNF inhibitor, XPro1595, and the non-selective TNF inhibitor, Etanercept (ETN) was examined in porcine pancreatic elastase (PPE) induced AAA mice, and findings with XPro1595 was confirmed in angiotensin II (ANGII) induced AAA in hyperlipidemic apolipoprotein E (*Apoe*) ^–/–^ mice.

**Results:**

XPro1595 treatment significantly reduced AAA expansion in both models, and a similar trend (*p* = 0.06) was observed in PPE-induced AAA in ETN-treated mice. In the PPE aneurysm wall, XPro1595 improved elastin integrity scores. In aneurysms, mean TNFR1 levels reduced non-significantly (*p* = 0.07) by 50% after TNF inhibition, but the histological location in murine AAAs was unaffected and similar to that in human AAAs. Semi-quantification of infiltrating leucocytes, macrophages, T-cells, and neutrophils in the aneurysm wall were unaffected by TNF inhibition. XPro1595 increased systemic TNF levels, while ETN increased systemic IL-10 levels. In ANGII-induced AAA mice, XPro1595 increased systemic TNF and IL-5 levels. In early AAA development, proteomic analyses revealed that XPro1595 significantly upregulated ontology terms including “platelet aggregation” and “coagulation” related to the fibrinogen complex, from which several proteins were among the top regulated proteins. Downregulated ontology terms were associated with metabolic processes.

**Conclusion:**

In conclusion, selective inhibition of solTNF signaling reduced aneurysm expansion in mice, supporting its potential as an attractive treatment option for AAA patients.

## Introduction

Abdominal aortic aneurysm (AAA) is an irreversible dilatation of the abdominal aorta that occurs in approximately 5% of elderly males (1). AAA progresses asymptomatically, but aneurysm rupture leads to acute intra-abdominal hemorrhage that is associated with up to 90% mortality (2). Current treatment options for AAA are limited to surgical intervention when AAA expansion reaches 55 mm in diameter in males (3), and no effective pharmacological alternative to slow AAA growth or prevent rupture is available (4). AAA pathophysiology is not fully understood, but local destruction of elastin and collagen in the aortic wall is considered an early event leading to the reorganization of extracellular matrix (ECM) composition (5). Disruption of the aortic wall structure releases elastokines which initiates an acute immunoreaction and infiltration of immune cells primarily dominated by macrophages, neutrophils, and T lymphocytes, releasing several pro-inflammatory cytokines, including tumor necrosis factor (TNF) (6–8). These cytokines stimulate macrophages and vascular smooth muscle cells (VSMCs) to release matrix metalloproteinases (MMPs), with MMP-2 and MMP-9 being the most critical ones in AAA progression (9, 10). MMP-9 derives primarily from infiltrating monocytes and macrophages, whereas MMP-2 derives primarily from activated vascular smooth muscle cells (11). Continued degradation of the ECM will eventually weaken the aortic wall, causing vascular smooth muscle cells to undergo apoptosis and thereby lead to thinning of the media (12), which ultimately results in AAA rupture.

Elevated TNF levels have been observed in both plasma and aneurysm wall samples from patients with AAA, suggesting that TNF plays a significant role in the pathogenesis of the disease (13–15). In support of this, a lack of functional TNF in mice has been shown to attenuate CaCl_2–_induced aneurysm development (16). TNF exists in two bioactive forms (17), but it is unclear whether both forms contribute to AAA development. TNF is synthesized as a transmembrane protein (tmTNF) (18) that is cleaved from the cell membrane by metalloproteinase TNF-converting enzyme (TACE/ADAM17) and released to the circulation and surrounding tissue as a soluble trimer complex (solTNF) (19). The effect of TNF is mediated by TNF receptor (TNFR) 1 and TNFR2, resulting in both overlapping and distinct biological outcomes (17). SolTNF has a higher affinity for TNFR1, and thus primarily drives a proinflammatory response through TNFR1 activation (20), whereas tmTNF acts in a paracrine fashion and mainly signal through TNFR2, resulting in tissue repair and regeneration as well as immune-regulating functions (21–24). Although the specific contributions of solTNF and tmTNF in AAA development remain uncertain, temporal systemic deletion of TACE prevents AAA formation in mice by attenuating inflammation and ECM disruption, indicating a crucial role of solTNF-TNFR1 signaling in AAA development (25). Thus, blocking solTNF is a promising therapeutic target for preventing AAA expansion.

Non-selective pharmacological inhibition of TNF using etanercept (ETN; a fusion protein consisting of TNFR2 attached to Fc of IgG that target both forms of TNF) has been effective in treating inflammatory diseases such as rheumatoid arthritis and inflammatory bowel disease (26). ETN is now recognized as an effective strategy in the management of chronic inflammation. Xiong et al. reported that Infliximab (another non-selective anti-TNF therapy) could inhibit aneurysm growth, attenuate elastic fiber disruption, and reduce macrophage infiltration in a CaCl_2_-induced AAA murine model (16), indicating that this strategy of TNF-antagonism may also apply to the treatment of AAA. It is uncertain, however, whether infliximab is effective in mice or whether the observed effects were mediated through off-target effects (27). Unfortunately, the use of non-selective TNF inhibition has been linked to rare but severe side effects, including increased susceptibility to serious infections (28, 29), potential risk of congestive heart failure (30), and demyelinating events (31, 32). Therefore, a new class of drugs called dominant-negative TNF (DN-TNF) inhibitors, which only inhibit solTNF signaling, has been developed to counteract the side effects of non-selective TNF therapy (33). The use of DN-TNF allows selective inhibition of the suggested proinflammatory solTNF-TNFR1 signaling pathway while preserving the suggested immunoregulatory and tissue-repairing functions of tmTNF-TNFR2 signaling, thereby hopefully diminishing the adverse effects of non-selective TNF treatment. Among the leading DN-TNFs today is the experimental drug XPro1595, which is a mutein of TNF that forms complexes with naïve solTNF. These complexes are incapable of binding to TNFRs, particularly TNFR1 (33, 34). XPro1595 treatment has successfully dampened several TNF-driven experimental diseases such as arthritis, Huntington’s disease, multiple sclerosis, spinal cord injury, and stroke (33, 35–38). We therefore hypothesize that selective inhibition of solTNF using XPro1595 will prevent AAA expansion by inhibiting the proinflammatory solTNF-TNFR1 signaling. This hypothesis was tested in two experimental murine AAA-models: 1) intraluminal porcine pancreatic elastase (PPE) infusion of the infrarenal aorta to test the effects of selective inhibition of solTNF by XPro1595 and non-selective inhibition of TNF by ETN on AAA expansion; and 2) chronic angiotensin II (ANGII) infusion in hyperlipidemic apolipoprotein E (*Apoe*)^–/–^ mice to test only the effect of XPro1595 on AAA expansion.

## Materials and methods

### Animals and human tissue

Experiments were performed according to an approved protocol by the Danish Animal Experiments Inspectorate (2015-15-0201-00474). Male C57BL/6J mice were purchased from Janvier Laboratories, Le Genest-Saint-Isle, France, and were allowed to acclimatize for 1 week prior to experiments. *Apoe*^–/–^ mice originated from Jackson Laboratory, United States, and were bred in house at the Biomedical Laboratory, University of Southern Denmark. Genotyping of the *Apoe*^–/–^ was done using primers (Sigma-Aldrich, Søborg, Denmark), with sequences provided by Jackson Laboratory. The experiments were performed at our facility where the mice were housed in a 12 h light/dark cycle, room temperature of 20°C, and air humidity of 55%. During the entire experiment, the animals had free access to food and tap water. Human aneurysm tissue and human ascending aortic punch specimens were collected after written consent and with approval from the Regional Committee on Health Research Ethics for Southern Denmark (S20140202 and M20080028).

### Induction of abdominal aortic aneurysm by perfusion of porcine pancreatic elastase (PPE)

Male C57BL/6J mice (9–10 weeks old) were anesthetized using a ketamine (Ketalar, Pfizer, Sandwich, Kent, United kingdom), and xylazine (Bayer Healthcare, Shawnee Mission, KS, United States) mixture and underwent laparotomy, followed by isolation of the abdominal aorta from the left renal vein to the iliac artery bifurcation. The baseline outer abdominal aortic diameter (OAD) during systole was measured using video recordings captured by a Nikon D3400 camera. The infrarenal aorta was then occluded using a silk suture (6.0, Amann, FST, Heidelberg, Germany) and a polyethylene catheter (4.0 Ethilon suture, Ethicon) was placed in the infrarenal aorta, allowing infusion of PPE (1.5 units/mL, Sigma-Aldrich, Søborg, Denmark) to expand the aorta to twice its size for 5 min. After removal of the catheter, the aortotomy was sutured with an 11.0 Ethilon suture (Ethicon), blood flow was reestablished, and the laparotomy was closed. Mice were given rimadyl (Zoetis, Farum, Denmark) for pain management. The health status and body weight of the mice were monitored daily for the following 14 days. On the day of surgery, mice were divided into three groups and treated by intraperitoneal injections (IP) of either XPro1595 [20 mg/kg, INmune Bio Inc, La Jolla, CA, United States, *n* = 14, (34)], etanercept (ETN, 20 mg/kg, Enbrel, Sandoz, Kundl, Austria, *n* = 14) or vehicle (physiological saline, Fresenius Kabi, Copenhagen, Denmark, *n* = 14) every third day during the next 14 days. At the end of the experiment, mice were re-anesthetized, the infrarenal aorta was isolated, and the final OAD was recorded. The percentage of increase in OAD (ΔOAD) was determined by an observer blinded to the treatment using the difference between baseline and final OAD of the infrarenal aorta. Collected organs were snap frozen in liquid nitrogen and stored together with EDTA-plasma at –80°C. Mice were terminated by perfusion with 10% normal formalin (Hounisen Laboratorieudstyr A/S, Skanderborg, Denmark) through the heart for 4 min at 120 mmHg pressure. For determination of abundant proteins in the aortic wall in the early phase of AAA expansion, a series of mice (vehicle *n* = 6; XPro1595 *n* = 7) were terminated 7 days post-surgery.

### Induction of abdominal aortic aneurysm by subcutaneous angiotensin II infusions in hyperlipidemic *Apoe* ^–/–^ mice

AAAs were induced in 8–12 weeks old male *Apoe*^–/–^ mice by chronic ANGII infusion *via* subcutaneous minipumps (Alzet model 2004, DURECT TM Corporation, Cupertino, CA, United States) releasing 60 μg/kg/hour ANGII (Calbiochem, Merck, Søborg, Denmark) for 28 days as previously described (39). In brief, pumps were implanted subcutaneously in the dorsal region of the mice through a neck incision under 3% isoflurane (IsoFlo^®^ vet, Orion Pharma, Nivå, Denmark) anesthesia. Mice were given *ad libitum* high-fat diet (RD Western diet D12079B, Research Diets Inc., Brogården, Hørsholm, Denmark) from 1 week before pump insertion and throughout the experiment. The mice were monitored daily and treated with XPro1595 (2 mg/kg, INmune Bio Inc., La Jolla, CA, United States *n* = 20 total, *n* = 14 completed) or vehicle (physiological saline, Fresenius Kabi, Copenhagen, Denmark, *n* = 19 total, *n* = 13 completed) by IP injections every third day for 28 days, starting on the day of surgery (day 0). Mice found dead during the experiment were analyzed for aortic rupture (12 died of aortic rupture, one died of unidentified reasons). Changes in inner aortic diameter were followed in anesthetized mice (3% isoflurane) by ultrasound using LOGIQ e portable ultrasound imaging system (GE Healthcare, Brøndby, Denmark) with a 22 MHz central frequency probe (L10-22-RS, GE healthcare, Brøndby, Denmark), starting on day 0 before surgery and then once a week for the following 4 weeks. Ultrasound measurements were determined by an observer blinded to the treatment.

Mice were terminated on day 28, and the aortas were fixed in 10% normal formalin (Hounisen Laboratorieudstyr A/S, Skanderborg, Denmark) for 24 h. The aortas were then placed on black wax Petri dishes and imaged with a Leica M80 dissecting microscope and a Leica IC80 HD digital microscope camera. The maximal outer abdominal aortic diameter and AAA surface area as a measure of aneurysm volume (surface area defined at a longitudinal piece of 7.7 mm to contain the full dilation of all aortas) were measured using Image J software (1.53a Wayne Rasband, National Institutes of Health, Bethesda, MA, United States). All measurements were performed by an observer blinded to treatment. The suprarenal abdominal aortas were embedded in paraffin for later histological analysis.

### Immunohistochemical staining

For immunohistochemical staining, human full wall AAA samples were collected during open surgical repair of growing AAAs, and ascending aortic wall punches were collected during coronary artery bypass surgeries. All human and murine tissues for histological analyses were fixed in 10% normal formalin (Hounisen Laboratorieudstyr A/S, Skanderborg, Denmark) and embedded in paraffin. Then, 5 μm sections were deparaffinized and hydrated in ethanol (99–75%) followed by demasking in either citrate buffer pH 6 (10 mM, Merck Søborg, Denmark) or TEG buffer pH 9 (10 mM, Tris Base, VWR, Søborg, Denmark). The sections were blocked for endogenous peroxidase activity in 3% H_2_O_2_ (Merck, Søborg, Denmark) in TBS and then blocked in 5% skimmed milk (Merck, Søborg, Denmark) or 3% bovine serum albumin (BSA, Sigma-Aldrich, Søborg, Denmark) in TBS containing 0.05% Tween20 (TBST, Sigma-Aldrich, Søborg, Denmark). Sections were then incubated overnight at 4°C with primary antibody directed against; CD45 (1:100, #550539, BD Pharmingen, Albertslund, Denmark), CD3 (1:200 Abcam, ab16669, Cambridge, United Kingdom), CD206 (1:1,000, Abcam, ab64693, Cambridge, United Kingdom), Ly6G (1:500, ab238132, Abcam, Cambridge, United Kingdom), fibronectin (1:300, ab268020, Abcam, Cambridge, United Kingdom), MMP-9 (1:200, ab38898, Abcam, Cambridge, United Kingdom), TNFR1 (1:500, ab19139, Abcam, Cambridge, United Kingdom), or TNFR2 (1:100, LS-B5301, LSBio, Copenhagen, Denmark). The following day, sections were incubated with appropriate horseradish-peroxidase (HRP)-conjugated secondary antibodies (1:1,000, DAKO, Agilent, Glostrup, Denmark) or with anti-rabbit EnVision+ System (DAKO, Agilent, Glostrup, Denmark), then washed in TBST. Positive staining was detected by applying diaminobenzidine (DAB, K3468, DAKO, Agilent, Glostrup, Denmark). Nuclei were counterstained using Mayer’s hematoxylin (Sigma-Aldrich). As negative controls, staining was tested on parallel sections of aortic aneurysmal samples by omitting the primary antibody or with the appropriate isotype IgG control (DAKO, Agilent, Glostrup, Denmark). All immunohistochemically stained tissue sections were analyzed by Olympus BX51 microscope, and images were captured by Olympus DP26 camera and analyzed in Image J. Cell counts of DAB positive cells (MMP-9, CD45, Ly6G and CD3) and % area of positive staining (TNFR1 and TNFR2) were measured based on means of duplicate AAA tissue sections for each mouse and expressed as the number of cells pr. mm^2^ AAA tissue or % positive staining, respectively. All analyses were done blinded. To assess elastin integrity, sections of aortic aneurysms were stained in 1% acidified Miller’s Elastin stain (Atom Scientific, Hyde, United Kingdom) according to the manufacturer’s instructions. The structural composition of elastin in the aneurysmal wall was semi-quantified by a scoring system described in Sun et al. (40).

### Measurement of cytokines, chemokines, and tumor necrosis factor receptor levels in aneurysm and plasma samples

Aneurysm tissues sampled 14 days after induction by the PPE model were lysed in Complete Mesoscale Lysis Buffer [10 mL 1x TRIS lysis buffer containing; 100 μL phosphate inhibitor cocktail #2 (Sigma-Aldrich, Søborg, Denmark), 100 μL phosphate inhibitor cocktail #3 (Sigma-Aldrich, Søborg, Denmark), and 1 tablet complete Mini, EDTA-free (Roche, Hvidovre, Denmark)] and homogenized by beads in a Tissue Lyzer II (Qiagen, Aarhus, Denmark) (Qiagen, Germany) for 3 min with maximum speed of 300 rpm and centrifuged for 20 min at +4°C at 12.000 × g. The supernatants were collected and stored at –80°C. Protein concentrations were determined using the Pierce BSA Protein Assay kit (Thermo Fischer, Roskilde, Denmark) (Bio-Rad, Copenhagen, Denmark). Cytokines and chemokines were then measured in aneurysmal protein samples and plasma samples using two different Mesoscale Discovery™ (MSD, Rockville, MA, United States) mouse pro-inflammatory V-Plex plus kits while TNFR1 and TNFR2 levels were determined using Ultra-Sensitive Kit (MSD, Rockville, MA, United States). Protocols were performed according to the manufacturer’s instructions. All samples were run in duplicate. Data were analyzed using MSD Workbench software. The cut-off threshold for inclusion of results was set at CV < 20%.

### Explorative mass spectrometry proteome analyses of XPro1595 and vehicle-treated abdominal aortic aneurysms at early development

Murine abdominal aorta aneurysms sampled 7 days after AAA induction by the PPE model were homogenized using a TissueLyser system (Qiagen, Aarhus, Denmark) with stainless steel beads (Qiagen, Aarhus, Denmark) in a lysis buffer [100 mM DTT, 5% sodium deoxycholate, 1% β-octylglucoside, 20 mM Tris, pH 8.8, supplemented with complete, Mini, EDTA-free Protease Inhibitor Cocktail Tablets and PhosSTOP (Roche, Hvidovre, Denmark)]. Protein samples were acetone-precipitated, re-dissolved in 0.2 M tetraethylammonium bicarbonate, and trypsinized overnight. Samples (4 μg tryptic peptides per sample) were randomly labeled with 11-plex tandem mass tags (TMT, Thermo Fisher Scientific, San Jose, CA, United States); mass tag 131°C was a pool of all AAA samples and served as internal control. Proteome data are protein abundances relative to the internal control. Mixed peptide samples were high pH fractionated as previously described (41). Nano-LC-MSMS of fractionated samples was performed on an Orbitrap Eclipse tribrid mass spectrometer (Thermo Fisher Scientific, San Jose, CA, United States) equipped with a nanoHPLC interface (Dionex UltiMate 3000 nano HPLC). The samples (5 μL) were loaded onto a custom-made fused capillary pre-column [2 cm length, 360 μm OD, 75 μm ID packed with ReproSil Pur C18 3 μm resin (Dr. Maish, GmbH)] with a flow of 5 μL/min for 7 min. Trapped peptides were separated on a custom-made fused capillary column (25 cm length, 360 μm OD, 75 μm ID, packed with ReporSil Pur C13 1.9 μm resin) using linear gradient ranging from 88 to 86% solution A (0.1% formic acid) to 27–32% B (80% acetonitrile in 0.1% formic acid) over 119 min. Mass spectra were acquired with an Orbitrap Eclipse Tribrid mass spectrometer with FAIMS Pro interface (Thermo Fisher Scientific, San Jose, CA, United States), switching between CVs of −50 and −70 V with 2 s cycle time. MS1 spectra were acquired at 60,000 resolutions with a scan range from 400 to 1,200 m/z, normalized AGC target of 100%, and maximum injection time of 50 ms. Precursors were filtered using monoisotopic peak determination set to peptide, charge state 2–4, dynamic exclusion of 60 s with ± 10 ppm tolerance excluding isotopes and different charge states, and a precursor fit of 70% in a window of 0.4 m/z for MS2 (50.000 resolution, normalized AGC target of 100 %, maximum fill time 86 of ms). All Eclipse raw data files were processed and quantified using Proteome Discoverer version 2.4 (Thermo Scientific, Waltham, MA, United States) as previously described (41).

Enrichment analysis was performed using R clusterProfiler 4.0 (arguments: ont = BP; nPerm = 10,000; minGSsize = 3; maxGSSize = 800; pvalueCutoff = 0.05; pAdjustMethod = “BH”) (42).

### Statistics

The primary endpoint of the study is the aneurysmal progression rate. In previous experiments, we observed a mean relative maximal aortic diameter increase of 127.3% ± 36.8 SD for the PPE-model (unpublished data). An aortic diameter difference of 30–33% is considered clinically significant. For a *t*-test with 5% significance and 80% power, each group needs 13 animals, when the ratio between intervened and control mice is 1:1. Therefore, we used 13–14 mice per group for the two experiments.

Statistical analyses and graphical representation of data were performed using GraphPad Prism version 6.0 (GraphPad Software, San Diego, CA, United States). All normally distributed data passing the D’Agostino and omnibus tests were analyzed by Student’s *t*-test for two-group comparisons, and significance of change between more than two groups was calculated using one-way analysis of variance (ANOVA) followed by Bonferroni’s *post-hoc* test for comparing means between groups or using two-way ANOVA with repeated measurements followed by Bonferroni’s *post hoc* testing. Data are represented as mean ± standard error of mean (SEM). Differences between non-Gaussian data not passing the normality test were analyzed by Mann-Whitney *U*-test and presented as median with interquartile range.

For normally distributed data, Grubb’s outlier test was used to identify outliers. Here, one sample in the XPro1595-treated-group in the ANGII model was identified as an outlier after analysis of both aortic diameters and surface area of AAAs and was excluded from the statistical analysis and all further downstream analyses. The sample is shown as a red square (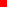) in the corresponding figures. A Fisher exact probability test with Freeman-Halton extension was used for a test of statistical significance for contingency table data of elastin degradation grade distributions. Explorative proteomic data were analyzed by unpaired *t*-test for each protein followed by *fdr* correction for multiple testing.

Comparisons with *P* < 0.05 were considered statistically significant and were denoted by asterisk(s).

## Results

### Selective inhibition of soluble form of tumor necrosis factor reduces expansion of elastase-induced abdominal aortic aneurysms

**FIGURE 1:**
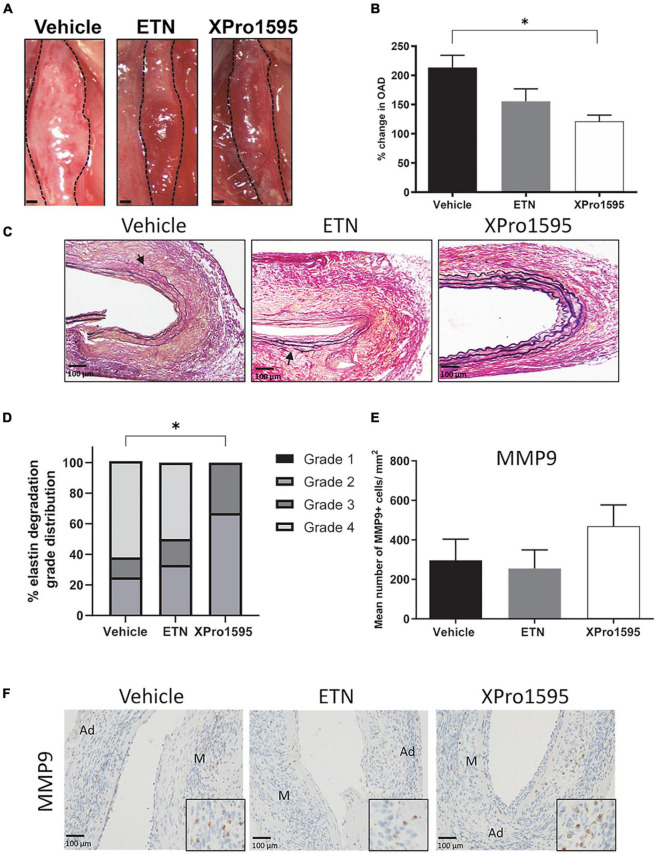
Selective inhibition of solTNF prevents expansion in PPE-induced AAA. **(A)** Representative macroscopic images displaying PPE-induced AAAs after 14 days of treatment with vehicle (saline), ETN (20 mg/kg), or XPro1595 (20 mg/kg). **(B)** *In vivo* AAA expansion measured as percentage change in the maximal outer diameter of the abdominal aorta (OAD) from day 0 to day 14 (*n* = 15–16). **(C)** AAA cross-sections stained with Miller’s elastic stain (black) after TNF inhibition (*n* = 3–5). Breaks in elastic fibers are marked with arrows. **(D)** Percentage distribution of elastic fiber integrity (grades 1–4) according to treatment groups (*n* = 3–5). **(E,F)** The number of MMP-9-positive cells/mm^2^ cross-sectional aneurysmal area from mice treated with vehicle-, ETN-, or XPro1595 (*n* = 3–5). Data are shown as mean SEM. * Indicates *p* < 0.05 analyzed by one-way ANOVA using Bonferroni test for multiple comparisons. Contingency table data in E was analyzed by Fisher exact probability test with Freeman-Halton extension. M, media; Ad, adventitia.

To determine whether TNF inhibition dampens elastase-induced AAA, mice were treated with the selective solTNF inhibitor, XPro1595, or with the non-selective TNF inhibitor ETN for 14 days. XPro1595 significantly reduced AAA expansion by 40% when compared to vehicle-treated control mice assessed by external outer abdominal aortic diameter (OAD) *in vivo* (Figures 1A,B). A similar, but non-significant, trend was seen with ETN treatment (*p* = 0.06, Figures 1A,B). TNF inhibition did not affect body weight or the relative mass of heart, liver, and kidneys to body weight (Supplementary Figures 1A,B).

### Selective inhibition of soluble form of tumor necrosis factor protects against elastic fiber degradation in the aneurysm wall

To address the disintegration of elastin in the aneurysm wall, elastin was stained using Miller’s elastin stain. In both the vehicle- and the ETN-treated group, the aneurysmal elastic lamellae were disorganized, stretched with areas containing multiple ruptured elastic fibers (Figure 1, arrows), while the elastin lamellae in the XPro1595-treated group were more organized with only few ruptured elastin lamellae (Figure 1C). Using a scoring system of elastin integrity described in Sun et al. (40), a significantly lower score was found in the XPro1595-treated mice than the vehicle-treated mice (Figure 1D). In contrast, elastin preservation was not observed after ETN treatment (*p* = 0.69, Figure 1D). The preserved elastin in the XPro1595-treated group was not caused by fewer MMP-9 positive cells/mm^2^ located within the aneurysm wall (Figure 1E), where MMP-9-positive cells were mainly associated with macrophage like cells in the adventitia (Figure 1F) that were evenly distributed between treatment groups (Figures 1E,F).

### Tumor necrosis factor receptor localization in the murine aneurysmal wall is comparable to that of humans

**FIGURE 2:**
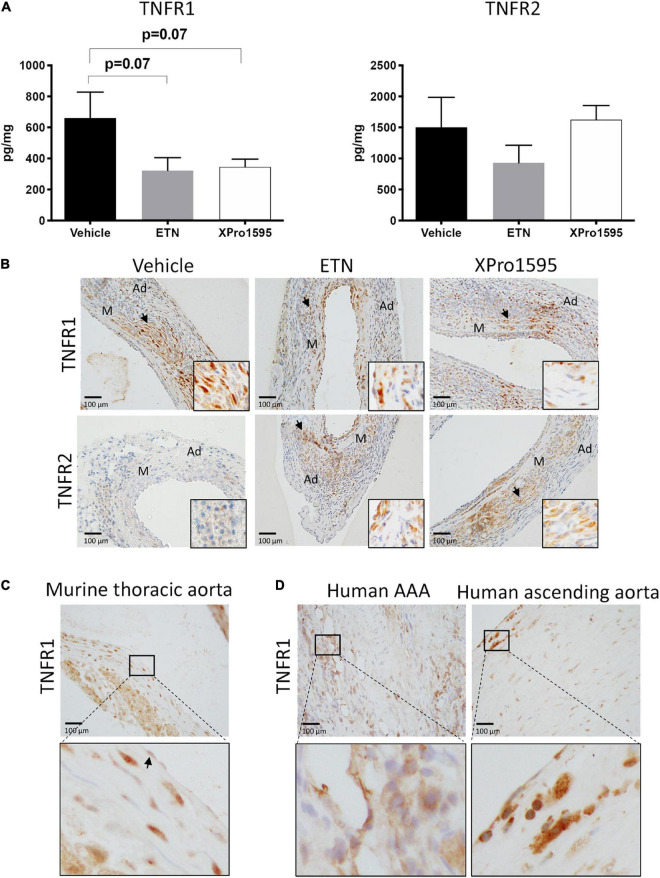
TNF receptor protein levels and receptor localization in murine and human abdominal aortic tissue. **(A)** Aneurysmal protein levels of TNFR1 and TNFR2 from PPE- induced aneurysm in mice treated with vehicle (saline), ETN (20 mg/kg), or XPro1595 (20 mg/kg) (*n* = 3–5). Data are shown as mean SEM. A non-significant trend was detected in TNFR1 levels by one-way ANOVA using Bonferroni test for multiple comparisons. **(B)** Representative distribution of TNFR1 and TNFR2 in the PPE-induced AAA wall after treatment with saline, ETN, or XPro1595 for 14 days (*n* = 3–5). **(C)** TNFR1 distribution in unchallenged thoracic murine aorta. **(D)** Distribution of TNFR1 in human AAA specimens (*n* = 2) and human ascending aortas (*n* = 2). Black arrowheads indicate positive stained cells. M, media; Ad, adventitia.

XPro1595 and ETN treatment resulted in a trend toward a 50% reduction in TNFR1 protein levels in the aneurysm wall when compared to vehicle-treated mice (*p* = 0.07, Figure 2A, left graph), while TNFR2 protein levels were unaffected by TNF inhibition (Figure 2A, right graph). This reduction in TNFR1 did not appear to be confined to a specific cell type in the aneurysm wall, as TNFR1 was localized in some VSMCs, infiltrating leukocyte-like cells, and endothelial cells with no apparent differences (Figure 2B, upper panel). TNFR2 was associated with leukocyte-like cells in the adventitia and some VSMCs and was unaffected by treatment (Figure 2B, lower panel). In thoracic mouse unchallenged aorta, TNFR1 immunolabeling was identified in most cells in media and adventitia, while in endothelial cells only a discrete labeling was observed (Figure 2C, arrow). The localization of TNFR1 in human AAA specimens and ascending aorta punches resembled that of murine aneurysms and thoracic aorta (Figure 2D). In the aneurysm wall, mainly leukocytes-like cells were found to be TNFR1-positive, while TNFR1 immunolabeling in human ascending aorta was associated with discrete staining in VSMCs, with more pronounced labeling in endothelial cells.

### Protection of abdominal aortic aneurysm expansion by soluble form of tumor necrosis factor inhibition is not associated with reduced leukocyte infiltration in the aneurysm wall

**FIGURE 3:**
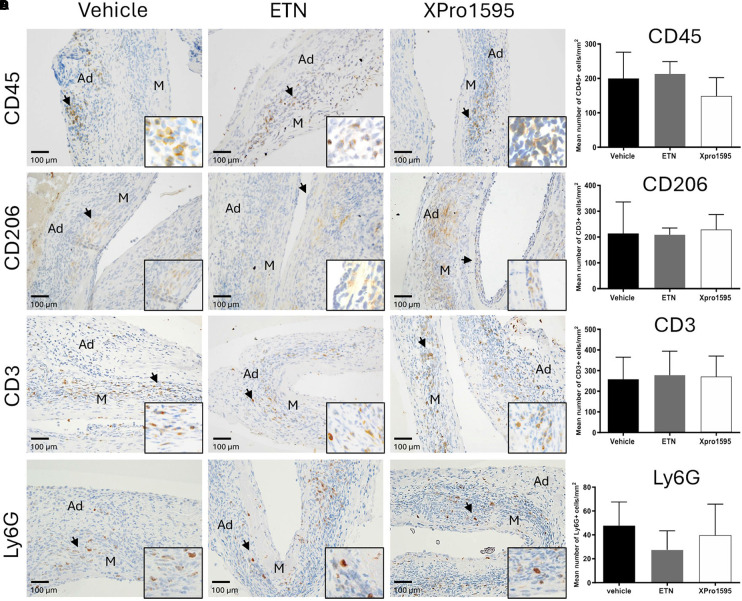
Effect of TNF inhibition on infiltrating immune cells 14 days after PPE-induced AAA formation. Representative micrographs after TNF inhibition showing the distribution of infiltrating immune cells (left) and semi-quantification (right, number of positive cells/mm^2^) of CD45-positive leukocytes **(A)**, CD206-positive M2-like macrophages **(B)**, CD3-positive T-cells **(C)**, and Ly6G-positive neutrophils **(D)** in the aneurysm wall after TNF inhibition (*n* = 3–5). Black arrowheads indicate positive stained cells. Data are shown as mean ± SEM. None of the semi-quantifications reached significance on one-way ANOVA using Bonferroni test for multiple comparisons. M, media; Ad, adventitia.

We observed no difference in the number of infiltrating CD45-positive leukocytes/mm^2^ in the aneurysm wall after TNF inhibition (Figure 3A). All groups displayed CD45-positive cells primarily in tunica adventitia and to lesser extent tunica media (Figure 3A, arrows). Also, the numbers of anti-inflammatory M2-like CD206-positive macrophages/mm^2^ (Figure 3B, arrows), of CD3-positive T-cells/mm^2^ (Figure 3C, arrows), and of Ly6G-positive neutrophil cells/mm^2^ (Figure 3D, arrows) were similar between treatment groups, and all positive staining was restricted to tunica adventitia (Figures 3B–D, arrows).

### Selective soluble tumor necrosis factor inhibition and non-selective tumor necrosis factor inhibition differentially regulate cytokine levels locally and systemically in porcine pancreatic elastase-induced abdominal aortic aneurysms mice

**FIGURE 4:**
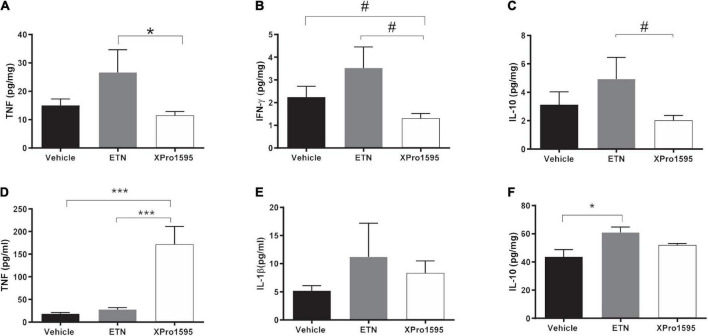
Pro- and anti-inflammatory cytokine profile systemically or locally produced in PPE-induced aneurysms after TNF inhibition. Changes in aneurysmal cytokine levels of TNF (*n* = 5–8) **(A)**, IFN-γ (*n* = 6–8) **(B)**, and IL-10 (*n* = 6–8) **(C)** 14 days after PPE-induced AAA mice treated with vehicle (saline), ETN (20 mg/kg), or XPro1595 (20 mg/kg). Changes in plasma levels of TNF (*n* = 7–8) **(D)**, IL-1β (*n* = 8) **(E)**, and IL-10 (*n* = 7–8) **(F)**. Data are shown as mean SEM. No significance was observed by one-way ANOVA using Bonferroni test for multiple comparisons. *P*-values are denoted *p < 0.05, ***p < 0.001, and ^#^ denotes *p* < 0.05 for unpaired Student’s *t*-test for two-group comparisons.

We next examined whether TNF inhibition affected local and/or systemic production of cytokines. Locally in the aneurysmal wall, there was a non-significant 50% increase in TNF protein levels in the ETN-treated AAAs when compared to vehicle-treated (*p* = 0.11), whereas TNF levels were significantly reduced in XPro1595-treated AAAs compared to ETN treatment (Figure 4A). A similar trend was seen with the pro-inflammatory cytokine interferon-gamma (IFNγ, Figure 4B) and the anti-inflammatory cytokine interleukin (IL)-10 (Figure 4C). No differences were observed in levels of IL-1β, IL-2, IL-4, IL-5, IL-6, IL-12p70, or KC/GRO in aneurysm tissue (Supplementary Table 1). In contrast to the aneurysm wall, there was a 10-times increase in plasma solTNF levels after XPro1595 treatment, whereas ETN treatment did not affect circulating solTNF levels (Figure 4D). Plasma levels of IL-1β (Figure 4E) and IL-10 (Figure 4F) were increased in ETN-treated mice, whereas the IL-10 levels in XPro1595-treated mice were similar to the levels in vehicle-treated mice. The levels of other measured circulating cytokines (IL-2, IL-4, IL-5, IL-6, KC/GRO) were not affected by TNF inhibition (Supplementary Table 2).

### Selective inhibition of soluble tumor necrosis factor increases fibrinogens and fibronectin in early abdominal aortic aneurysms

**FIGURE 5:**
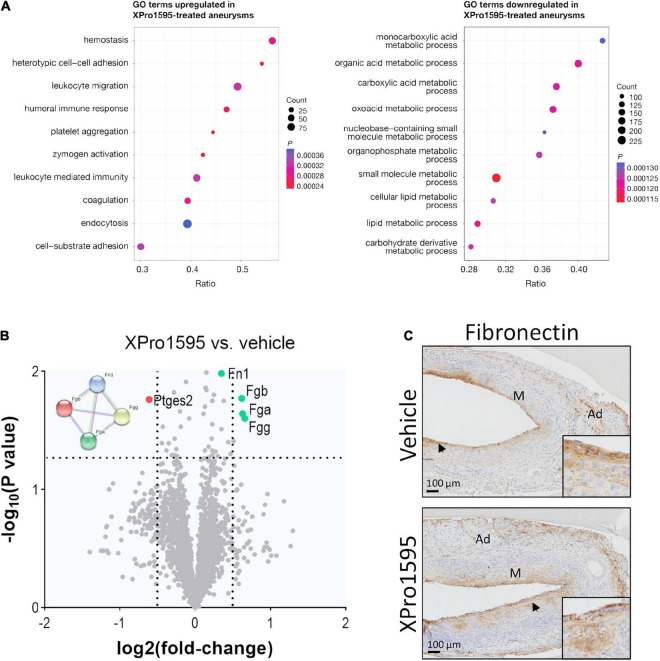
The fibronectin/fibrinogen complex is upregulated in aneurysms after inhibition of solTNF by XPro1595**. (A)** Dot plots show the top 10 enriched biological function terms from proteins up- (left) or down-regulated (right) in PPE-induced aneurysms from XPro1595-treated mice (*n* = 7) vs. vehicle (*n* = 6) at day 7. Dot size indicates number of proteins found to be regulated by XPro1595 for a given term. The ratio shows the proportion of a functional term covered by proteins regulated by XPro1595, and the dot color indicates the level of significance. **(B)** Volcano plot of aneurysmal proteins and their differential expression in aneurysms from XPro1595-treated mice vs. vehicle. Horizontal dashed line denotes a 0.05 cut-off *p*-value, and vertical dashed lines denote a log2(fold-change) of 0.5 in either direction. Fibronectin (FN1), fibrinogen chains FGA, FGB, and FGG and their protein-protein interactions are illustrated by use of the STRING resource (74), showing interactions determined experimentally (pink lines), from curated databases (blue lines), and/or from text-mining (yellow lines) (left upper corner of volcano plot). **(C)** Fibronectin contents in representative abdominal aortic aneurysmal tissue sections from mice infused with elastase and treated with XPro1595 (20 mg/kg) or vehicle (*n* = 3–5). Black arrowheads indicate positive stained cells. Proteomics data were analyzed by unpaired *t*-test for each protein followed by *fdr* correction for multiple testing. M, media; Ad, adventitia.

To explore the effect of TNF inhibition in initial stages of AAA development, aneurysms were examined by mass spectrometry 7 days after disease onset. None of the 5,469 identified proteins were significantly regulated by XPro1595 treatment after adjustment for multiple testing (see Supplementary Table 3 for a complete list of identified proteins). However, enrichment analysis revealed ontologies including “hemostasis,” “platelet aggregation,” and “coagulation” to be significantly upregulated by XPro1595 treatment (Figure 5A). In addition, significantly upregulated ontologies included “leukocyte migration,” “zymogen activation,” and “leukocyte-mediated immunity,” while downregulated ontologies covered a variety of metabolic processes (Figure 5A). In particular, three fibrinogen chains (FGA, FGB, and FGG) and fibronectin (FN1), which are key players in platelet aggregation and coagulation biological processes, were among the top regulated proteins shown in the volcano plot (green colored dots in Figure 5B) while prostaglandin E2 synthase (MPGES2) was among the most downregulated proteins after XPro1595 treatment (Figure 5B, red colored dot). Immunohistochemistry showed fibronectin in intima draining into media (Figure 5C, arrows) in the aneurysm wall in both vehicle- and XPro1595-treated mice.

### Selective inhibition of soluble tumor necrosis factor inhibits abdominal aortic aneurysm expansion in angiotensin II-treated *Apoe* ^–/–^ mice

**FIGURE 6:**
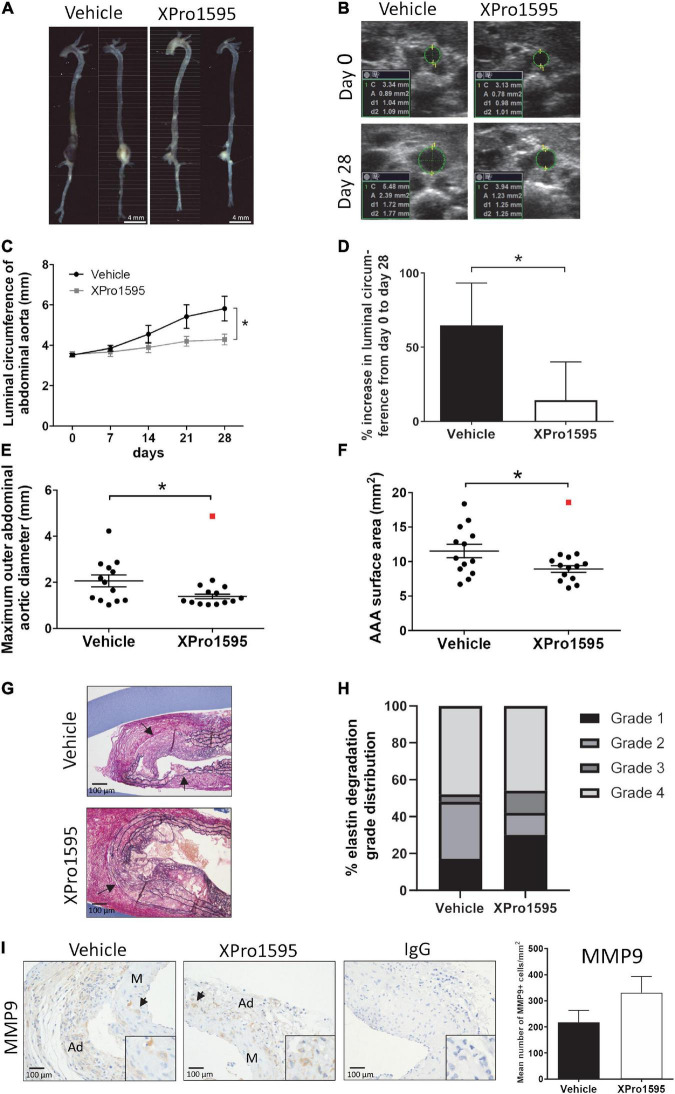
Inhibition of solTNF prevents ANGII-induced AAA expansion. **(A)** Representative macroscopic images showing AAA expansion in vehicle (saline) or XPro1595 (2 mg/kg) ANGII-infused *Apoe*^–/–^ mice. **(B)** Representative ultrasound images of the abdominal aorta at its maximal inner circumference at day 0 and day 28 of the ANGII-induced AAA from vehicle-treated and XPro1595-treated mice. **(C)** ANGII-induced AAA expansion assessed by inner abdominal aortic circumference. **(D)** The percentage change in the maximal inner abdominal aortic circumference from day 0 to day 28 in vehicle- and XPro1595-treated mice assessed by ultrasound€ **(E)** *Ex vivo-*measured maximal outer aortic diameter in the two groups. **(F)** *Ex vivo*-measured surface area of the AAA treated with vehicle and XPro1595. **(G)** Representative micrographs of AAA cross-sections stained with Miller’s elastin stain (black) in vehicle- and XPro1595-treated AAAs. Arrows point to rupture of elastic fibers. **(H)** Medial elastin degradation semi-quantified by a 4-grade system in aneurysms from XPro1595- and vehicle-treated mice. **(I)** MMP-9 immunoreactivity in cross-sections of vehicle- and XPro1595-treated aneurysms and the representative semi-quantification (number of positive cells/mm^2^). Black arrowheads indicate positive stained cells. IgG isoform was used as negative control. Results are shown as mean SEM if data passed the D’Agostino and Pearson omnibus normality tests. Mann–Whitney test was applied in D and presented as median with interquartile range as data were non-normally distributed. *Denotes *p* < 0.05 analyzed by unpaired student *t*-test for two-group comparison or two-way ANOVA with Bonferroni test for multiple comparisons. Contingency table data in H was analyzed by Fisher exact probability test with Freeman-Halton extension. Outliers were identified by Grubb’s outlier test for normally distributed data and indicated by red squares (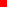) and excluded from the statistical analyses. M, media; Ad, adventitia.

As seen with the PPE-induced aneurysms, XPro1595 significantly attenuated AAA progression in ANGII-induced AAA according to different parameters of aneurysmal size (Figure 6). Inner luminal aortic circumference measured by ultrasound revealed a significant reduction in the relative luminal size at day 28 when measured as circumference of the inner aorta (Figures 6A–C). Also, the percentage increase from days 0 to 28 in the aortic luminal circumference was smaller in the XPro1595-treated group (Figure 6D). The abdominal aorta distal to the renal artery branch was used as a control reference point for aortic expansion, and no difference in percentage circumference increase (day 0 to post-surgery day 28) was observed between groups (vehicle: 1.17 ± 5.8% vs. XPro1595: 4.43 ± 6.16%, *n* = 13–14, *p* = 0.70). Similarly, external AAA *ex vivo* measurements of maximum outer abdominal aortic diameter and surface area of AAAs were significantly decreased in mice receiving XPro1595 (Figures 6E,F). The decrease in AAA size after selective inhibition of solTNF by XPro1595 was not associated with changes in body weight and organ-to-body weight ratios of heart, spleen, liver, and kidneys were unaffected by treatment (Supplementary Figures 1C,D). Despite a decrease in aneurysm expansion, the survival rate of mice treated with XPro1595 (70.0%) was similar to that of vehicle-treated mice (68.4%). Six mice in each group died between experimental days 2 and 26; all died from either AAA rupture or rupture of the ascending aorta, except one in the vehicle group that died from unknown causes.

### Inhibition of soluble tumor necrosis factor in angiotensin II-induced abdominal aortic aneurysms did not affect elastic fiber degradation nor immune cell infiltration

**FIGURE 7:**
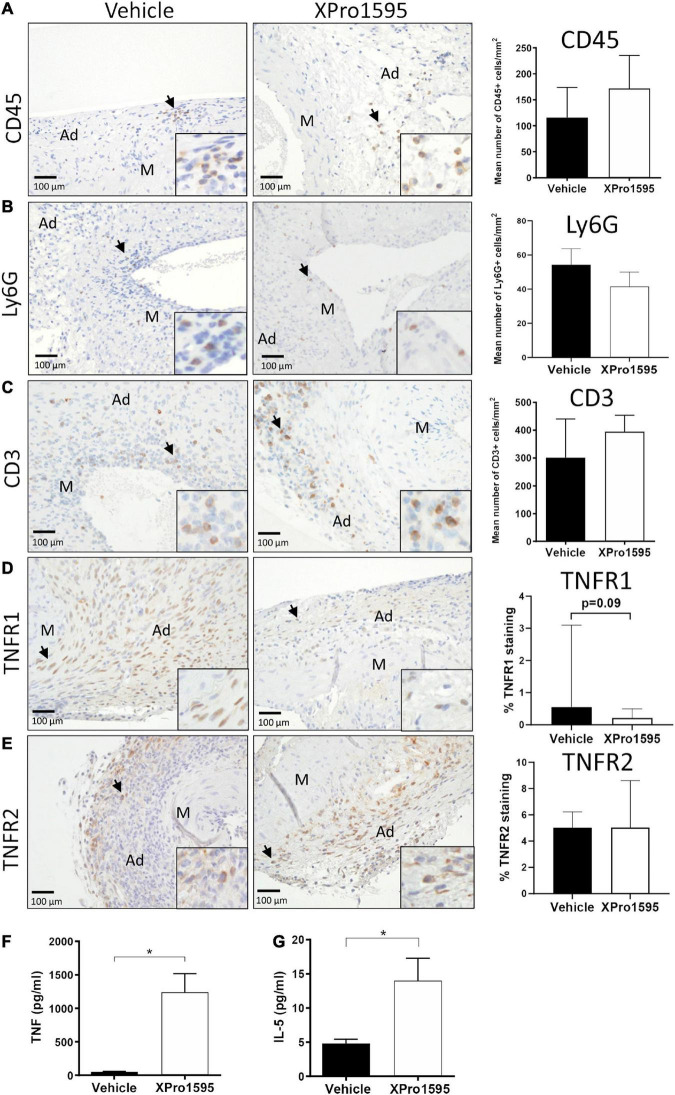
Characterization of aneurysm wall composition after inhibition of solTNF in ANGII-induced abdominal aortic aneurysms. Representative micrographs of CD45-positive leukocytes **(A)**, Ly6G-positive neutrophils **(B)**, CD3-positive T cells **(C)** (*n* = 13), TNFR1 localization **(D)**, TNFR2 localization **(E)** (*n* = 6) and their respective semi-quantification (number of positive cells/mm^2^ or % positive staining) in cross-sections of ANGII-induced AAA treated with vehicle or XPro1595 (2 mg/kg). Black arrows point to examples of positive stained cells. Circulating TNF plasma levels (*n* = 4–5) **(F)** and IL-5 (*n* = 6–7) **(G)** in ANGII-induced AAA treated with vehicle or XPro1595. Data are shown as mean SEM if data passed the D’Agostino and Pearson omnibus normality tests **(B,C,E–G)**. *Denotes *p* < 0.05 analyzed by unpaired Student’s *t*-test for normally distributed data, otherwise by Mann-Whitney test represented as median with interquartile range **(A,D)**. M, media; Ad, adventitia.

In the ANGII model, inhibition of solTNF did not have any apparent effect on elastin integrity (Figures 6G,H). Around 50% of the aneurysms in both groups were associated with severe destruction of elastic fibers (indicated by arrows in Figure 6G) and thereby reached an elastin degradation score of grade 4. As in the elastase induced aneurysms (Figures 1E,F), the mean number of MMP-9-positive cells/mm^2^ in aneurysmal tissue was unaltered after XPro1595 treatment compared to vehicle-controls (Figure 1I). The MMP-9-positive cells were abundant in tunica adventitia of the aneurysm, supposedly associated with inflammatory cells of this layer, but MMP-9-positive cells were also present in the tunica media and assumed to be in VSMCs (Figure 6I, arrows). In line with above results, the CD45-positive infiltrating leukocytes (Figure 7A, arrows), Ly6G-positive infiltrating neutrophils (Figure 7B, arrows), and CD3-positive T-cells (Figure 7C) were primarily localized to tunica adventitia and to a smaller extent in tunica media, and their numbers/mm^2^ were not affected by XPro1595. TNFR1-positive cells were primarily limited to fibroblast-like cells in tunica adventitia of the aneurysmal wall (Figure 7D, arrows) and TNFR1 aneurysmal contents showed a trend toward a 60% reduction after selective TNF inhibition with XPro1595 (Figure 7D, graph), as also seen in the elastase induced aneurysms immunolabeling of TNFR2-positive cells was detected in leukocyte-like cells in tunica adventitia (Figure 7E, arrows), but no difference in the levels of TNFR2 were detected after selective TNF inhibition (Figure 7E, graph). In this AAA model TNF plasma levels were also significantly increased (24 times) in XPro1595-treated ANGII infused mice (Figure 7F). Circulating plasma levels of the T-cell associated cytokine IL-5 were also significantly elevated (Figure 7G), while plasma levels of IL-1β, IL-2, IL-4, IL-6, IL-10, and KC/GRO did not differ between treatment groups (Supplementary Table 1).

## Discussion

The present study aimed at elucidating whether treatment with the selective solTNF inhibitor XPro1595 could prevent AAA expansion by dampening the inflammatory response mediated *via* TNFR1 signaling. We found that selective inhibition of solTNF by XPro1595 reduced AAA development in both the PPE aneurysm model and ANGII-induced aneurysms in hyperlipidemic *Apoe* ^–/–^ mice. This inhibitory effect on aneurysm growth was more pronounced than non-selective inhibition of TNF by ETN treatment, where we observed a similar but non-significant 40% reduction of maximal outer aortic diameter. Others have previously shown that inhibition of TNF by gene deletion reduces CaCl_2_-induced aneurysm growth (16). The underlying mechanism was ascribed to decreased inflammatory response and diminished MMP-2 and MMP-9 activity, thereby resulting in the preservation of the elastin lamellae structure. It is widely accepted that dysregulation of TNF adversely affects elastin by directly suppressing elastin mRNA levels in aortic smooth muscle cells (43) and increases expression of elastin-degrading enzymes MMP-2 and MMP-9 in aneurysmal tissue (16). In the present study, the underlying mechanism of solTNF inhibition also resulted in more intact and organized elastic lamellae when compared to both ETN-treatment and controls. This indicates that the degrading effects on elastin are mediated by solTNF. However, we did not detect differences in the distribution of MMP-9 in the aneurysm wall. This does not rule out a potential reduction in MMP-9 activity by XPro1595 treatment as reported by others using non-selective TNF inhibitors (16, 44, 45). Future studies are needed to address this point.

As mentioned earlier, solTNF is released from cells by cleavage of tmTNF by the metalloprotease-disintegrin TACE (46). As TACE is elevated in human AAAs, local high levels of solTNF are assumed to be present in growing AAAs and to drive inflammatory processes in the aneurysm wall (25). These findings are supported by *ex vivo* experiments where the release of solTNF, TNFR1 and TNFR2 correlated with the levels of TACE in aortic lesions of *Apoe*^–/–^ mice (47). SolTNF, not tmTNF, is thought to be the main factor in the growth of AAA. This is based on the discovery of genetic variants in the promoter region of TACE that raise the risk of AAA development (47, 48). Therefore, the net effect of TACE inhibition is expected to be like XPro1595 inhibition, as both interventions prevent solTNF binding to TNFRs. In line with our results, Kaneko et al. (25), showed that global knockdown of TACE inhibited AAA expansion by reducing the inflammatory response and thereby preserving elastin lamellae integrity in the aneurysm wall.

In the present study, local cytokines in the aneurysms were differentially affected by selective solTNF inhibition. We observed that TNF levels locally in the aneurysm wall tended to be increased after ETN treatment, but this increase was significantly lower in the XPro1595-treated mice. A similar pattern was seen for IFNγ and IL10, indicating that solTNF inhibition reduced the inflammatory response in the aneurysm wall. It is well established that TNF and IFNγ synergistically promote leukocyte entry into the aortic wall by activating endothelial cells to express the intercellular adhesion molecule 1 (ICAM1) (49). This may explain why the protective effect of ETN on AAA development is less pronounced compared to XPro1595 treatment. Although we did not observe any apparent difference in infiltrating macrophages, M2-like macrophages, T-cells, or neutrophils in the aneurysm wall at the end of the experiments, it remains to be seen whether delayed entry of infiltrating leukocytes in XPro1595-treated mice can explain part of XPro1595’s protective role against AAA expansion. The local changes in cytokine levels were not reflected in circulating levels. In both AAA models, there was a 10–24-times increase in circulating TNF after XPro1595 treatment. However, since XPro1595 is a TNF mutein that forms trimer complexes with native TNF, it is expected that plasma solTNF levels will increase as our ELISA assay detects both active and inactive TNF-XPro1595 trimer complexes. This might also partly explain our previous findings of elevated circulating TNF in unchallenged mice treated with XPro1595 (50). Furthermore, loss of solTNF signaling due to XPro1595 treatment increases *Tnf* mRNA levels in brain tissue 1 and 3 days after permanent middle cerebral artery occlusion (51), which indicates that loss of solTNF signaling promotes TNF expression. Elevated levels of IL-10 were detected systemically only after ETN inhibition, which is in line with previous studies showing that not only the expression of IL-10 in macrophages and T-cells is increased after ETN treatment, but also their secretion (52, 53). Thus, inhibition of both forms of TNF has systemic anti-inflammatory effects mediated *via* IL-10. Surprisingly, circulating levels of IL-5, which is primarily produced by mast cells and T-helper 2 cells (54, 55), increased in the ANGII AAA model after XPro1595 treatment. Others have reported that IL-5 potentiates AAA expansion by directly or indirectly upregulating MMP-2 and MMP-9 expression (56); this does not correspond with our findings, although perhaps some of the expected beneficial effects of XPro1595 were masked by elevated circulating IL-5 levels.

TNF signaling in the vascular wall involves most cell types with TNFR1 expressed in endothelial cells, VSMCs, immune cells, and fibroblasts, as shown here and by others (57). As solTNF has a higher binding affinity for TNFR1, and tmTNF has a higher binding affinity for TNFR2, the effect of solTNF will mainly be mediated *via* TNFR1 activation (20, 58), while tmTNF-TNFR2 signaling may be more pronounced when solTNF is inhibited. TNFR1 signaling contributes significantly to the formation of intracranial aneurysms and to coronary aneurysm formation in association with Kawasaki Disease (59, 60). In the present study, selective TNF inhibition reduced aneurysmal TNFR1 levels non-significantly by 50–60% depending on the AAA mouse model. A general reduction of TNFR1 in the aneurysm wall could be explained by increasing TACE activity (25). In *Tnf^–/–^* mice, dendritic cells display a higher TACE expression (61). Also, circulating levels of TNFRs has been shown to be elevated in patients with thoracic aortic aneurysm and in patients with ruptured AAA compared to patients undergoing elective surgery (62), which correlates well with elevated TACE activity. The apparent reduction of TNFR1 compared to controls could be explained by the loss of solTNF signaling that dampens TNFR1 expression (63). The distribution of TNFR1 in the aneurysm wall did not appear to be associated with a reduction in any specific cell type in the aneurysm wall. TNFR2 levels and distribution in the aneurysm wall were unaffected by TNF inhibition, which raises the question of whether the trend toward reduced TNFR1 aneurysm levels was caused solely by changes in TACE activity.

The balance between M1- and M2-like macrophages plays an important role in AAA progression (64). In our experiments, there was no obvious difference in the number of CD206-positive M2 cells infiltrating the wall of the aneurysms. To our surprise, leukocyte infiltration was indifferent after TNF inhibition even though TNF signaling increases ICAM1 expression in endothelial cells, allowing leukocyte entry into the aortic wall (63, 65). Inhibition of solTNF signaling potentially reduced ICAM1 expression in endothelial cells and thereby leukocyte entry to the damaged aortic wall at the initiation of AAA expansion (days 3–7), which could partly explain the reduced AAA formation in the XPro1595-treated mice. Another likely possibility for the protective effects of XPro1595 could be a blood pressure-lowering effect. TNF inhibition by ETN has been reported to have no or a slightly lowering effect on blood pressure (66). It has further been reported that chronic infusion of XPro1595 does not alter mean arterial blood pressure or heart rate 2–4 weeks after spinal cord injury in mice (67). We did not detect any between-group differences in heart-to-body ratio in either model, which suggests that no pronounced differences in arterial blood pressure were present.

Because fibrinogen complex, fibronectin, and fibrinogens are elevated in XPro1595 in the early stages of aneurysm formation, it is possible that they initially support the fibro-protective effects of the damaged aortic wall. Fibrinogen deposition in the aortic wall may trigger an autoimmune response that could lead to activation of the complement alternative pathway and AAA progression (68). In human AAAs, increased deposition of pro-thrombosis fibrin/fibrinogen complexes within the AAA wall has been identified, along with an association of circulating fibrin/fibrinogen degradation products (D-dimers) and AAA growth or perhaps more likely, luminal thrombus formation (69–71), all believed to augment AAA progression. Thus, it is not clear from the present study whether the elevated fibrin-fibrinogen complex in AAA development after solTNF inhibition is associated with profibrotic stabilization of the AAA wall or simply indicates delayed AAA development in comparison to vehicle treatment.

In our study we did not detect any signs of toxicity by using XPro1595. XPro1595 treatment in both AAA models, did not affect changes in body weights or organ to body weight ratios of heart, kidney, and liver. Furthermore, XPro1595 is a mutated form of the human protein of TNF which makes the trimer complex unable to bind to TNFRs (33) and is therefore a natural pharmaceutical substance. We did not examine whether liver steatosis is present in XPro1595-treated mice with ANGII induced AAA. However, others have shown that XPro1595 can improve diet induced obesity (high-fat high-carbohydrate diet) and central-peripheral insulin impairment, in wild type mice partly through dampening of hepatic lipocalin-2, which is associated with and elevated in liver steatosis. These alterations suggest reduced risk of diet induced late onset of Alzheimer’s disease (72). Furthermore, we have previously shown in healthy, adult WT mice that long term XPro1595 treatment (2 months) in contrast to ETN treatment did not alter neurogenic zones and impair spatial learning and memory (50). These findings suggest that selective inhibition of SolTNF with XPro1595 is non-toxic in the tested doses. Furthermore, human clinical trials using XPro1595 in patients with mild Alzheimer’s disease (ClinicalTrials.gov Identifier: NCT05318976) and in patients with mild cognitive impairments (ClinicalTrials.gov Identifier: NCT05321498) have been successful and is currently in phase 2, thus has also passed toxicity testing in humans. Like in AAA patients, both of these diseases meet the criteria of high peripheral inflammation based on elevated blood levels of the inflammatory biomarker high-sensitivity c-reactive protein (hs-CRP) (73).

In conclusion, inhibition of solTNF prevents AAA expansion by a mechanism involving the preservation of elastin lamellae. These findings support that targeting the solTNF-TNFR1 signaling pathway would be an attractive treatment strategy and potentially even better than non-selective TNF therapy for patients with growing AAA, which is highly desired.

## Data availability statement

The mass spectrometry proteomics dataset presented in this study is publicly available. The data can be found below: http://www.proteomexchange.org/, PXD035178.

## Ethics statement

The studies involving human participants were reviewed and approved by the Regional Committee on Health Research Ethics for Southern Denmark (S20140202 and M20080028). The patients/participants provided their written informed consent to participate in this study. The animal study was reviewed and approved by The Danish Animal Experiments Inspectorate (2015-15-0201-00474).

## Author contributions

JS, JL, and KL designed the study. SG, EG, EF, ML, SB-M, HB, MO, and LS performed the experiments, data analyses, and interpret the results. LR and JL provided the human samples. SG, EG, and JS drafted the manuscript. All authors critically reviewed, edited, and approved the final version of the manuscript.

## Supplementary material

Supplementary Figure 1 Increase in body weight and organ-to-body weight ratio at the endpoint of either PPE (0–14 days)- or ANGII treatment (0–28 days), showing the body weight difference **(A,C)** and the organ-to-body weight ratios **(B,D)**. (PPE: vehicle *n* = 14; ETN *n* = 14; XPro1595 *n* = 14); (ANGII: vehicle *n* = 13; XPro1595 *n* = 14). The data are shown as mean ± SEM. No significance was observed by one-way ANOVA using Bonferroni test for multiple comparisons or unpaired Student’s *t*-test for normally distributed data.

Supplementary Table 1 Table of cytokine levels measured in AAA homogenate but not affected by TNF inhibition treatment in PPE animals (*n* = 4–8). The data are shown as mean ± SEM. No significance was observed by one-way ANOVA using Bonferroni test for multiple comparisons.

Supplementary Table 2 Table of cytokine levels measured in plasma but not affected by TNF inhibition treatment in PPE (*n* = 6–8) and ANGII-treated animals (*n* = 13–14). The data are shown as mean ± SEM. * Indicates *p* < 0.05 analyzed by one-way ANOVA using Bonferroni test for multiple comparisons or unpaired Student’s *t*-test for normally distributed data. ^§^ Denotes data already represented in Figures 5E,F. ¤ Denotes data already represented in Figure 7G.

Supplementary Table 3 Complete list of identified proteins in the abdominal aortic wall by mass spectrometry 7 days after PPE-induced AAA in mice. Mice were treated with XPro1595 (20 mg/kg) (*n* = 7) or vehicle (*n* = 6).
