# Corrigendum: Selective inhibition of soluble tumor necrosis factor signaling reduces abdominal aortic aneurysm progression

**DOI:** 10.3389/fcvm.2024.1466614

**Published:** 2024-08-19

**Authors:** Silke Griepke, Emilie Grupe, Jes Sanddal Lindholt, Elizabeth Hvitfeldt Fuglsang, Lasse Bach Steffensen, Hans Christian Beck, Mia Dupont Larsen, Sissel Karoline Bang-Møller, Martin Overgaard, Lars Melholt Rasmussen, Kate Lykke Lambertsen, Jane Stubbe

**Affiliations:** ^1^ Department of Cardiovascular and Renal Research, Institute of Molecular Medicine, University of Southern Denmark, Odense, Denmark; ^2^ Elite Research Centre for Individualized Medicine in Arterial Diseases (CIMA), Odense University Hospital, Odense, Denmark; ^3^ Department of Cardiothoracic and Vascular Surgery, Odense University Hospital, Odense, Denmark; ^4^ Department of Clinical Biochemistry and Pharmacology, Odense University Hospital, Odense, Denmark; ^5^ Department of Neurobiology, Institute of Molecular Medicine, University of Southern Denmark, Odense, Denmark; ^6^ Department of Neurology, Odense University Hospital, Odense, Denmark; ^7^ BRIDGE—Brain Research—Inter-Disciplinary Guided Excellence, Department of Clinical Research, University of Southern Denmark, Odense, Denmark

**Keywords:** cardiovascular disease, abdominal aortic aneurysm, tumor necrosis factor inhibitor, translational research, vascular inflammation

A Corrigendum on Selective inhibition of soluble tumor necrosis factor signaling reduces abdominal aortic aneurysm progression By Griepke S, Grupe E, Lindholt JS, Fuglsang EH, Steffensen LB, Beck HC, Larsen MD, Bang-Møller SK, Overgaard M, Rasmussen LM, Lambertsen KL and Stubbe J (2022). Front. Cardiovasc. Med. 9:942342. doi: 10.3389/fcvm.2022.942342

**Error in Figure/Table**

In the published article, there was an error in Figure 3B as published. The same micrograph in Figure 3B as overview micrograph for both the vehicle and XPro1595 treated group stained with CD206. It is only in the representative presentation for each group it appears and does not affect the semi-quantification of CD206 between groups as they were performed on all sections from each individual mouse in the groups and does therefore not affect the overall results or conclusions**.** The corrected Figure 3 and its caption appear below.

**Figure 3:**
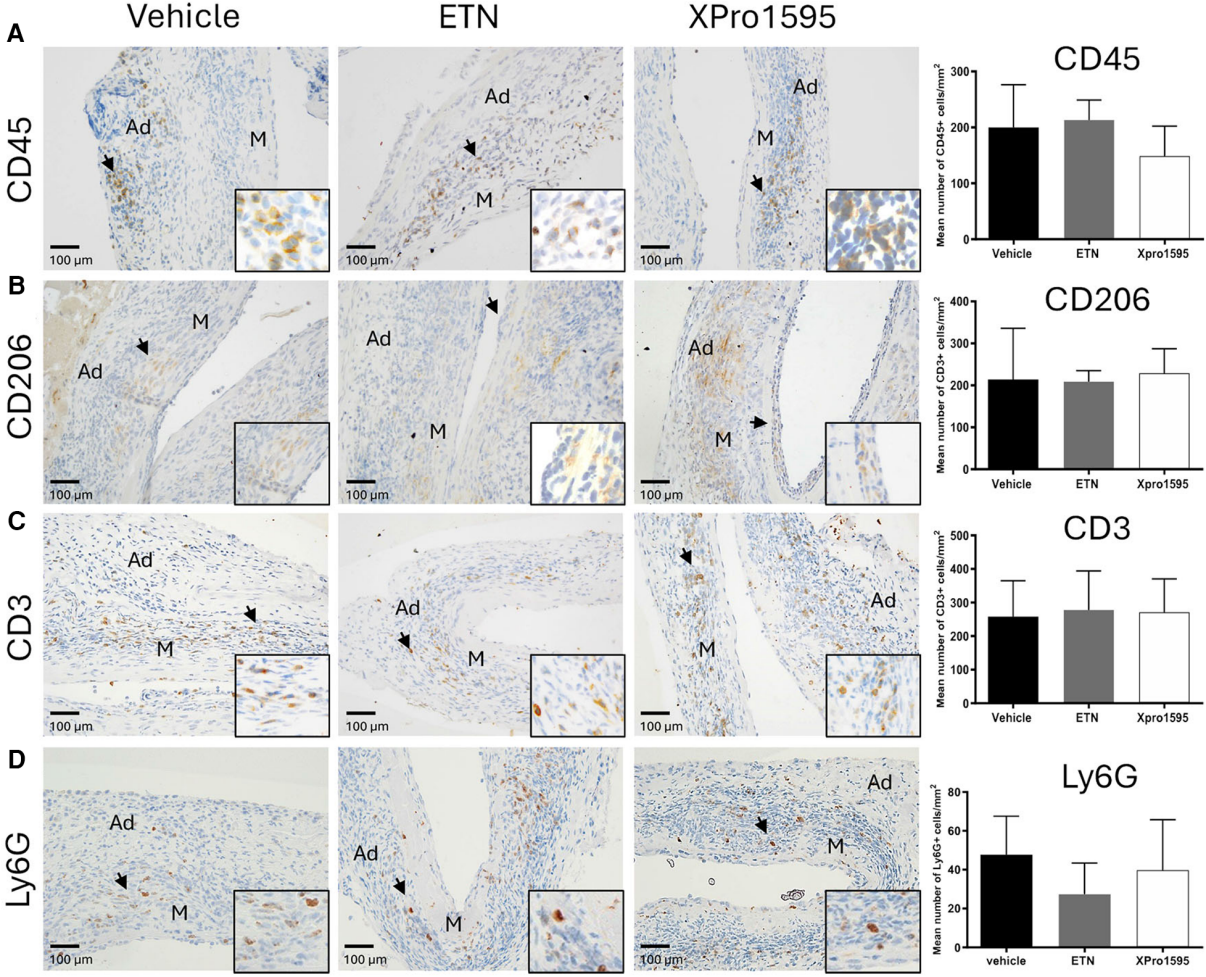
Effect of TNF inhibition on infiltrating immune cells 14 days after PPE-induced AAA formation. Representative micrographs after TNF inhibition showing the distribution of infiltrating immune cells (left) and semi-quantification (right, number of positive cells/mm^2) of CD45-positive leukocytes **(A)**, CD206-positive M2-like macrophages **(B)**, CD3-positive T-cells **(C)**, and Ly6G-positive neutrophils **(D)** in the aneurysm wall after TNF inhibition (*n* = 3–5). Black arrowheads indicate positive stained cells. Data are shown as mean ± SEM. None of the semi-quantifications reached significance on one-way ANOVA using Bonferroni test for multiple comparisons. M, media; Ad, adventitia.

The authors apologize for this error and state that this does not change the scientific conclusions of the article in any way. The original article has been updated.

