# Supplementary figures and images for: Selective inhibition of soluble tumor necrosis factor signaling reduces abdominal aortic aneurysm progression
Source: Front Cardiovasc Med. 2022 Sep 16;9:942342. doi: 10.3389/fcvm.2022.942342 (PMC9523116; doi:10.3389/fcvm.2022.942342)

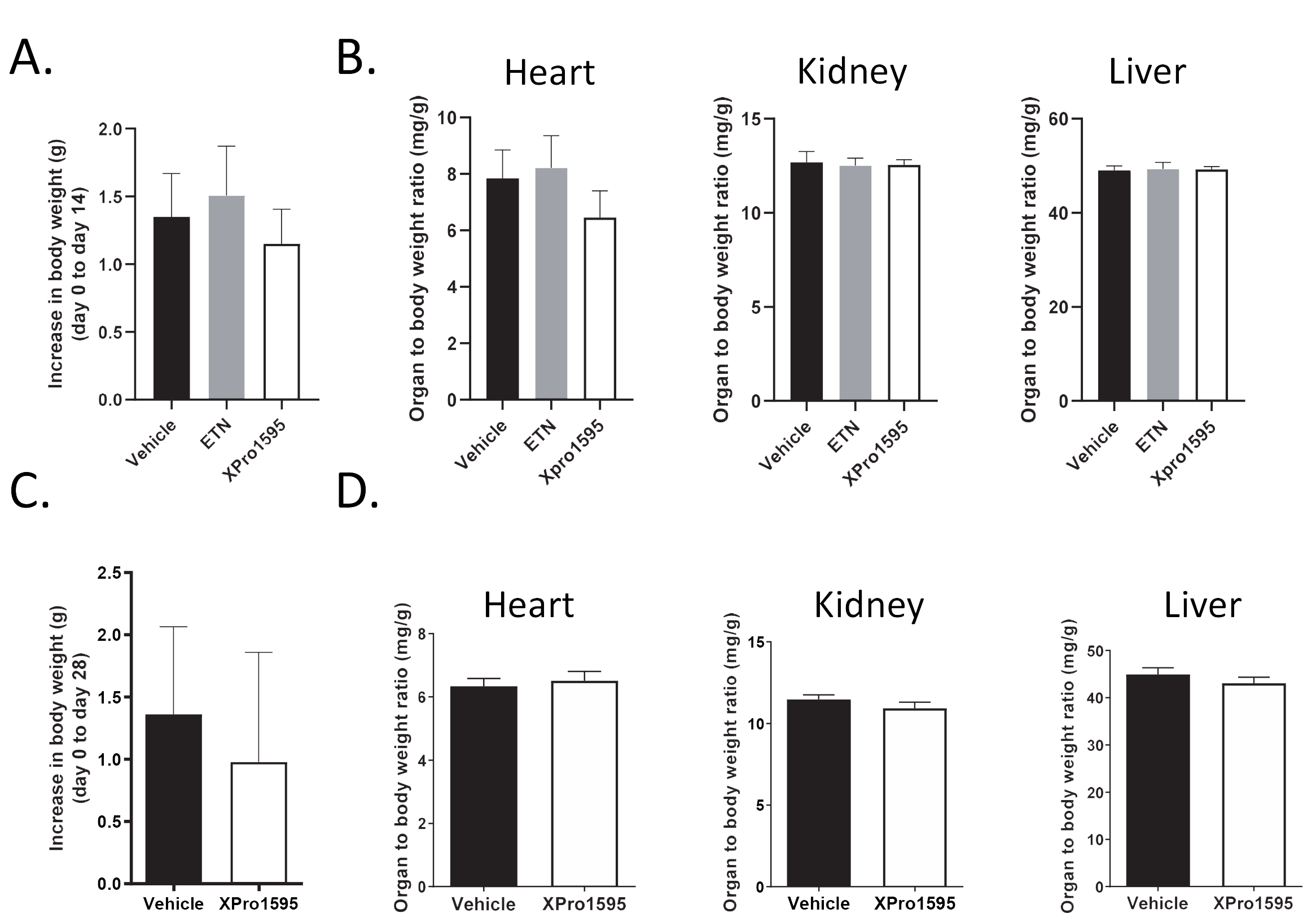

Supplement: Supplementary Figure 1 — Increase in body weight and organ-to-body weight ratio at the endpoint of either PPE (0–14 days)- or ANGII treatment (0–28 days), showing the body weight difference (A,C) and the organ-to-body weight ratios (B,D). (PPE: vehicle n = 14; ETN n = 14; XPro1595 n = 14); (ANGII: vehicle n = 13; XPro1595 n = 14). The data are shown as mean ± SEM. No significance was observed by one-way ANOVA using Bonferroni test for multiple comparisons or unpaired Student’s t-test for normally distributed data. [file Image_1.TIF]
