# Supplementary material for: Selective inhibition of soluble tumor necrosis factor signaling reduces abdominal aortic aneurysm progression
Source: Front Cardiovasc Med. 2022 Sep 16;9:942342. doi: 10.3389/fcvm.2022.942342 (PMC9523116; doi:10.3389/fcvm.2022.942342)
Supplement: Supplementary Table 1 — Table of cytokine levels measured in AAA homogenate but not affected by TNF inhibition treatment in PPE animals (n = 4–8). The data are shown as mean ± SEM. No significance was observed by one-way ANOVA using Bonferroni test for multiple comparisons. [file Table_1.DOCX]

Supplemental table 1

|  | **PPE MOUSE MODEL** | | |
| --- | --- | --- | --- |
| **CYTOKINES/CHEMOKINES** | Vehicle | ETN | XPro1595 |
| AAA homogenate | Mean value (pg/mg) | | |
| **IL-1β** | 79.0±21.58 | 54.23±23.30 | 98.29±43.20 |
| **IL-2** | 0.82±0.14 | 1.26±0.41 | 0.98±0.22 |
| **IL-4** | 0.74±0.16 | 0.67±0.12 | 0.68±0.08 |
| **IL-5** | 0.90±0.16 | 1.23±0.35 | 0.99±0.12 |
| **IL-6** | 642.9±241.6 | 681.4±226.8 | 474.0±151.1 |
| **IL-12p70** | 7.81±1.82 | 6.070±1.81 | 8.36±2.25 |
| **KC/GRO** | 145.5±32.94 | 130.3±36.25 | 153.1±43.55 |
