# Supplementary material for: Selective inhibition of soluble tumor necrosis factor signaling reduces abdominal aortic aneurysm progression
Source: Front Cardiovasc Med. 2022 Sep 16;9:942342. doi: 10.3389/fcvm.2022.942342 (PMC9523116; doi:10.3389/fcvm.2022.942342)
Supplement: Supplementary Table 2 — Table of cytokine levels measured in plasma but not affected by TNF inhibition treatment in PPE (n = 6–8) and ANGII-treated animals (n = 13–14). The data are shown as mean ± SEM. * Indicates p < 0.05 analyzed by one-way ANOVA using Bonferroni test for multiple comparisons or unpaired Student’s t-test for normally distributed data. § Denotes data already represented in Figures 5E,F. ¤ Denotes data already represented in Figure 7G. [file Table_2.docx]

Supplemental table 2

|  | **ANGII APOE^-/-^ MOUSE MODEL** | | **PPE MOUSE MODEL** | | |
| --- | --- | --- | --- | --- | --- |
| **CYTOKINES/CHEMOKINES** | Vehicle | XPro1595 | Vehicle | ETN | XPro1595 |
| Plasma | Mean value (pg/mL) | | Mean value (pg/mL) | | |
| **IL-1β** | 0.88±0.15 | 1.1±0.23 | *5.20±0.90***§** | *11.23±5.95***§** | *8.36±2.12***§** |
| **IL-2** | 3.4±0.39 | 3.1±0.67 | 2.4±0.82 | 2.4±0.34 | 2.3±0.32 |
| **IL-4** | 0.39±0.17 | 0.23±0.12 | 0.19±0.041 | 0.32±0.059 | 0.34±0.078 |
| **IL-5** | *4.82±0.62***¤** | *14.00±3.27***¤*** | 4.0±0.34 | 3.7±0.53 | 3.4±0.51 |
| **IL-6** | 144.5±63.96 | 166.7±49.43 | 42.6±7.10 | 41.1±11.0 | 66.9±15.21 |
| **IL-10** | 56.6±2.33 | 66.6±8.99 | *43.63±5.11***§** | *60.94±3.85***§*** | *52.13±0.96***§** |
| **KC/GRO** | 404±70.35 | 569.8±157.8 | 249.5±40.02 | 246.7±74.46 | 166.6±21.59 |
